# Occupational Exposure to Flour Dust. Exposure Assessment and Effectiveness of Control Measures

**DOI:** 10.3390/ijerph17145182

**Published:** 2020-07-17

**Authors:** Andrea Martinelli, Fabiola Salamon, Maria Luisa Scapellato, Andrea Trevisan, Liviano Vianello, Rosana Bizzotto, Maria Angiola Crivellaro, Mariella Carrieri

**Affiliations:** 1 Department of Cardiac Thoracic Vascular Sciences and Public Health, University of Padova, 35128 Padova, Italy; fabiola.salamon@unipd.it (F.S.); marialuisa.scapellato@unipd.it (M.L.S.); andrea.trevisan@unipd.it (A.T.); mariaangiola.crivellaro@aopd.veneto.it (M.A.C.); mariella.carrieri@unipd.it (M.C.); 2 SPISAL, Azienda ULSS7 Pedemontana, 36061 Bassano del Grappa (VI), Italy; liviano.vianello@aulss7.veneto.it; 3 SPISAL, Azienda ULSS6 Euganea, 35128 Padova, Italy; rosana.bizzotto@aulss6.veneto.it

**Keywords:** Flour dust, occupational exposure, control measures

## Abstract

The adverse effects associated with exposure to flour dust have been known since the 1700s. The aim of the study was to assess the occupational exposure to flour dust in Italian facilities, identify the activities characterized by the highest exposure, and provide information to reduce workers’ exposure. The study was performed in different facilities such as flourmills (*n* = 2), confectioneries (*n* = 2), bakeries (*n* = 24), and pizzerias (*n* = 2). Inhalable flour dust was assessed by personal and area samplings (*n* = 250) using IOM (Institute of Occupational Medicine) samplers. The results showed personal occupational exposure to flour dust over the American Conference of Governmental Industrial Hygiene (ACGIH) and the Scientific Committee on Occupational Exposure Limit (SCOEL) occupational limits (mean 1.987 mg/m^3^; range 0.093–14.055 mg/m^3^). The levels were significantly higher for dough makers in comparison to the dough formers and packaging area subjects. In four bakeries the industrial hygiene surveys were re-performed after some control measures, such as installation of a sleeve to the end of pipeline, a lid on the mixer tub or local exhaust ventilation system, were installed. The exposure levels were significantly lower than those measured before the introduction of control measures. The exposure level reduction was observed not only in the dough making area but also in all bakeries locals.

## 1. Introduction

Occupational exposure to flour dust is related to the onset of allergopathias such as intermittent or persistent asthma, conjunctivitis, rhinitis, and contact dermatitis [1,2,3,4,5,6,7]. These adverse effects associated with exposure to flour dust have long been known and are well documented [8]. Occupational asthma (OA) in bakers was first described in the 1700s by Ramazzini in his treatise *De Morbis Artificium Diatriba* [9]. In the United Kingdom, exposure to flour dust is the second most recognized cause for the onset of OA (38.8 per 100,000) [10]. Similarly, high rates of baker’s asthma have been reported in other European countries [11,12,13,14,15,16]. In Italy, a study aimed at investigating the awareness of Italian allergologists regarding OA, revealed that in the many cases (37.5%) of patients with OA were bakers [17].

The environmental concentration necessary to sensitize the exposed by inducing the triggering of symptoms has been still not demonstrated. An increased prevalence of respiratory symptoms has been reported at dust exposures of 1.5–4.0 mg/m^3^, with sensitization to flour dust being reported after exposures lower than 0.5 mg/m^3^ [18,19].

Most occupational exposure limits (OEL) for flour dust have been established as inhalable dust, and the OELs range from 0.5 to 10 mg/m^3^ [20]. In Italy, in absence of a national OEL, it is common to refer to the occupational limits (Threshold Limit Value-Time-Weighted Average, TLV-TWA), established by American Conference of Governmental Industrial Hygienist (ACGIH), that, since 1999, for flour dust, adopted a TLV-TWA of 0.5 mg/m^3^ and, from 2014, added the notation senR (respiratory sensitizer) [21].

In Europe, the Scientific Committee on Occupational Exposure Limits (SCOEL) recommended a OEL of 1 mg/m^3^ [22]. The SCOEL, as a general rule, did not recommend health-based limit values for sensitizing substances but, in view of the large number of exposed workers and the relatively large database, recognized that an exposure value of less than or equal to 1 mg/m^3^ of inhalable dust should protect the majority of exposed workers.

However, it should be taken into account that exposure levels <1 mg/m^3^ can trigger symptoms in already sensitized workers and that the sensitization of the respiratory tract appears at lower exposure levels, hence an OEL that protects all workers cannot be identified [23,24].

The aim of the study was to assess the occupational exposure to flour dust in Italian facilities, identify the activities characterized by the highest exposure, and provide information to reduce workers’ exposure.

## 2. Materials and Methods

### 2.1. Study Design

The study was carried out in northern Italy and was performed in three phases: assess flour dust exposure in different facilities (Phase 1); in a specific sector identify the activities characterized by the highest exposure levels and suggest control measures in order to reduce the exposure (Phase 2); and verify the effectiveness of the adopted measures (Phase 3). During Phase 1, industrial hygiene surveys were carried out in two flourmills, three bakeries, two confectioneries, and two pizzerias. Subsequently in the bakery sector, where the larger number of workers complained respiratory symptoms, additional 21 facilities (nine craft bakeries and 12 industrial bakeries), were investigated. The studied facilities were selected randomly between those present in Padova province (Veneto Region). Their characteristics are summarized in Table 1. The production processes were similar in all bakeries: dough making, dough forming, and packaging, but with different levels of automation. The daily flour consumption and the flour handling were different in the two type of bakeries. In the craft bakeries the daily flour consumption was below 500 kg (range from 63 kg to 500 kg) and the method of pouring the flour in the mixer tub, was mainly manual. In the industrial bakeries, the daily flour consumption was significantly higher than in the craft bakeries, in the range from 350 kg to 4000 kg, and the pouring of flour in the mixer tub was by a pipeline from the silos. In some cases, at the end of the pipeline, a cotton sleeve was installed.

**Figure 1:**
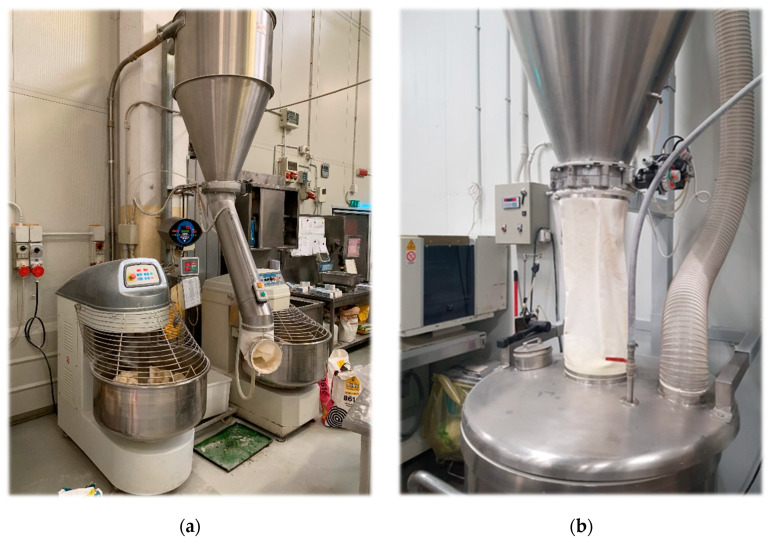
Installation of a sleeve at the end of pipeline (**a**); installation of a lid and a local exhaust ventilation system on a mixer tub (**b**).

During Phase 3, four bakeries among the 24 investigated were selected. The criteria selection was the adoption of preventive measures after the results of the surveys carried out in Phase 2. In these bakeries a new survey was performed in the same position and on the same subjects in order to evaluate the effectiveness of these measures. In particular, the control measures, reported in Figure 1, were: installation of a sleeve to the end of pipeline (bakeries 1 and 3), installation of a lid on the mixer tub (bakery 2), installation of a lid on the mixer tub and local exhaust ventilation system (bakery 4).

### 2.2. Dust Sampling and Analysis

Personal and area air samplings were performed. Inhalable flour dust in the air was sampled by IOM samplers equipped with PVC (polyvinyl chloride) filters (25 mm diameter, 5 μm pore size) connected with a SKC XR5000 pump (SKC Inc., Eighty Four, PA, USA) with constant flow calibrated at 2 L/min. A total of 250 samples were collected in the 30 facilities investigated (Phase 1: 58 samples; Phase 2: 163 samples; Phase 3: 29 samples). Personal samplings (*n* = 117) were performed in the worker breathing zone and area samplings (*n* = 133) came from samplers on tripods at a height of 160 cm above floor level. Samplings were carried out during all work activities and were lasted 4 h. Gravimetric determination of the dust was carried out by weighing the filters before and after sampling using a XPR6UD5 microbalance with a detection threshold of 0.0005 mg (Mettler Toledo, Columbus, OH, USA). Filters were acclimatized prior to weighing for 48 h in an Aquaria Climatic hood (Aquaria, Milan, Italy) with constant temperature and humidity (20 ± 1 °C; 50 ± 5%, respectively) to ensure standard weighing conditions. The limit of detection (LOD) for the flour dust was 0.01 mg, calculated using the average weight difference of the blank filters plus three times the standard deviation. None of the samples collected had values below the LOD.

### 2.3. Statistical Analysis

Statistical analysis was carried out using the StatsDirect version 2.7.7 (StatsDirect Ltd., Merseyside, UK). A non-normal distribution of all variables was observed by the Shapiro–Wilk test. Differences between groups were assessed using non-parametric tests: a Mann–Whitney test to compare two groups and a Kruskal–Wallis test to compare more than two groups. A Conover–Iman test was performed as a “post-hoc” test after Kruskal–Wallis. The comparison between pre- and post-introduction of control measures was assessed by Wilcoxon’s signed ranks test. Correlations between variables were assessed by linear regression analysis. In all tests, a *p*-value lower than 0.05 (two-tailed) was considered as statistically significant.

## 3. Results

The flour dust exposure levels found in the different facilities during Phase 1 are shown in Table 2. The exposure levels were, on the average, higher than the TLV-TWA proposed by ACGIH with exposure peaks being up to 14 times higher. The lowest exposure levels were detected in pizzerias. In these facilities the personal exposures were statistically lower than those measured in bakeries (*p* = 0.0110), where the highest mean personal exposure levels were found. Although the exposure levels in the bakeries were higher than the other facilities, no statistically significant differences were found in comparison with the levels measured in flourmills and confectioneries.

**Figure 2:**
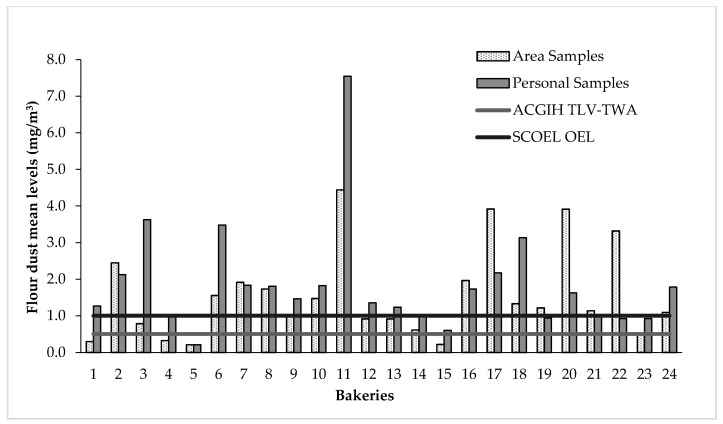
Mean flour dust levels in the 24 Italian bakeries. (ACGIH TLV-TWA, American Conference of Governmental Industrial Hygienist Threshold Limit Value-Time-Weighted Average; SCOEL OEL, Scientific Committee on Occupational Exposure Limits Occupational Exposure Limit).

The flour dust exposure levels found in the 24 bakeries are shown in Table 3. The results are described according by bakery type, daily flour consumption, and pouring method. Significantly higher values were found in the personal samples than in the area samples (*p* < 0.0001). The levels of flour dust in the 105 area samples ranged between 0.043–16.763 mg/m^3^, with 34.3% of them being above the SCOEL OEL and 54.3% being above the ACGIH TLV-TWA. The levels of personal exposure to flour dust were ranged between 0.148–14.055 mg/m^3^, with 65.5% of them being above the SCOEL OEL and 83.3% being above the ACGIH TLV-TWA (Figure 2). A statistically significant correlation (r = 0.72; *p* < 0.0001) was found between the personal exposure to dust and the dust pollution measured by the area samples in the working area of subjects (data not shown).

No statistically significant differences were found within bakery type or daily flour consumption, although according to the median, higher levels of dust were found in craft bakeries than in industrial (*p* = 0.09). On the contrary, statistically significant differences were found in relation to the pouring method of flour into the mixer tub. The use of a pipeline with a sleeve significantly reduced both the personal exposure and the dust pollution in comparison with the use of the pipeline without a sleeve or the manual method (*p* < 0.0001). These results were confirmed also when only the data (both area and personal samples) in dough making area were taken in account (data not shown).

Occupational exposure to flour dust was evaluated also according to the workers’ job task: dough maker, dough former, and packaging area employee (Table 4). The data showed statistically significant higher exposure levels for dough makers in comparison to the dough formers and packaging area subjects (*p* = 0.0011 and *p* < 0.0001, respectively). A statistically significant difference was also found between dough formers and packaging area subjects (*p* = 0.0085). Similar results were found in relation to the dust pollution measured in those areas.

Table 5 shows the results of Phase 3 and the comparison with the data measured before the installation of control measures described previously.

The exposure levels measured during Phase 3 were lower than the exposure levels measured in the same four bakeries before the introduction of control measures (Phase 2). The mean exposure level reduction during Phase 3 was observed not only in the dough making area (2.377 vs. 4.864 mg/m^3^), but also in all bakery locals (1.715 vs. 3.352 mg/m^3^). The reduction was statistically significant in both cases (*p* = 0.042 and *p* = 0.0298, respectively).

## 4. Discussion

Exposure to wheat flour dust can be considered inevitable, direct, and repetitive in the workplaces investigated. The results of environmental monitoring in all workplaces investigated showed personal occupational exposure to flour dust within the range 0.093–14.055 mg/m^3^ with a mean value equal to 1.987 mg/m^3^ (Phases 1 and 2). These values were comparable or lower than those found in other studies carried out in different countries in the last decade [25,26,27,28]. In the bakeries, as expected, the job tasks characterized by the higher exposure levels were the dough maker and the dough former. The results underline the need of control measures in order to reduce the exposure, especially during these activities, and avoid the sensitizations of workers. Indeed, some authors report an increased risk of rhinitis at inhalable dust concentrations higher than 1 mg/m^3^ and an increased risk of asthma at concentrations higher than 3 mg/m^3^ [29]. On the other hand, exposure levels lower than 0.5 mg/m^3^ seem reduce the rate of sensitization and the likelihood of symptoms in subjects already sensitized [30].

A significant reduction of the exposure levels could be achieved through some engineering intervention on the dough making area. The pouring of flour into the mixer tub seems to be a crucial step. Very high levels of flour dust were measured during this activity [31]. Some authors suggest the use of a pipeline connected to a silo in place of the manual pouring method that involves the shaking of bags producing a spread of dust. The installation of the silos with a pipeline, particularly advantageous also for the reduction of the risk of biomechanical overload of the spine and upper limbs in particular on the shoulders, has not proved to be equally effective for the reduction of the levels of dust [32].

The data of this study showed that the use of a pipeline without a sleeve could be ineffective to reduce the exposure. Indeed, the exposure levels in bakeries where the silos and pipelines without sleeves were installed, were comparable to those measured in the bakeries where the manual pouring method was used. On the contrary the installation of a pipeline with a sleeve significantly decreased the level of dust and consequently the exposure of workers. The mean exposure levels in the bakeries where this method was used were about 3–4 times lower than those measured in the other bakeries (both stationary samples and personal samples). The effectiveness of the use a pipeline with a sleeve was also confirmed by the data from the four bakeries evaluated during Phase 3 after the introduction of control measures. In particular, in the bakeries 1 and 3, where the only introduced control measure was the installation of sleeve, a reduction of exposure levels around 16% and 45% was observed, respectively (data not shown). The placement of a sleeve to the end of pipeline is an inexpensive but very effective modification, especially where silos and pipelines are already installed.

The installation of a lid on the mixer tub and a local exhaust ventilation system are further, but more costly, stages of primary prevention in this production sector. In this study, the highest reduction took place in the bakery 4 (about 70%) where these control measures were adopted.

Although these measures are very effective, there is a certain resistance to adopt them because of the impossibility for the dough maker to check the quality of the dough visually and also by touch. The use of a transparent screen or partially grilled screens seems a recommended compromise solution. Currently the lids of mixer tubs are opaque hence they do not allow viewing dough and because of this not many bakeries install them.

The decreasing of exposure levels could be obtained by modifying the behavior of workers and the working practices. Simple procedures such as to empty the bags of flour without shaking them, pouring the flour into the water and not vice versa, or cleaning the workplace using a vacuum cleaner in place of bristle brooms seem to be effective to reduce the exposure [33,34]. Moreover, to reduce the speed of action of the mixer, especially in the first 5 min when flour and water are not yet sufficiently mixed and part of the flour is not yet wet, could be another tip to reduce the dust flour exposure and consequently the onset of adverse symptoms. The speed should be reduced even after each addition of flour [35].

In relation to the dough processing operations, dusting flour on the work surfaces is another delicate operation, which is repeated numerous times during the working day. Some authors suggest the use of divider oils in place of sprinkling flour [3,34,36]. In the bakeries involved in the current study, only some of these procedures were adopted. In particular, in most of them the use of broom was common and in all of them the workers refused to use the oil because they reported a change of the flavor of products. In this case the use of large flours specially separated (so-called “round” or “passing” flour), such as rice flour or corn flour, could be recommended. In addition, the use of corn flour in place of wheat flour seems to reduce the sensitizations of workers because of its low allergenic power [37].

An important step in order to reduce the exposure levels is represented by the training of workers [34,38,39]. Indeed, activities such as dry sweeping and flour dusting by hand are still undertaken by the majority of bakeries, may be due that either to a limited knowledge of good working practices or that these were overlooked by both employers and employees.

It should also be noted that the exposure to flour dust in bakeries is also characterized by frequent short-term peak exposures lasting a few minutes (30 s–4 min) [37]. These peaks contribute not only to time-weighted average exposure but can play an important role in the advancement of awareness [31]. Since these exposure peaks correspond to relatively well-defined operations, the use of respiratory protection during these activities should be taken in account. The use of respiratory protective equipment has been demonstrated that can reduce the exposure and therefore may prevent the asthma onset [40].

## 5. Conclusions

Baker’s asthma is one of the most common types of asthma of a professional origin linked to exposure to flour dust and allergens contained in it. The exposure assessment to flour dust in the monitored workplaces highlighted exposure levels over the occupational exposure limits, especially in bakeries during the activities of dough making and forming. In these facilities the introduction of silos and pipelines as a pouring method of flour into the mixer tub does not seem to be effective without a placement of a sleeve at the end of the pipeline. This simple and inexpensive adjustment could reduce the exposure levels of workers up to 45%.

Levels of flour dust exceeding the occupational exposure levels were found also in flourmills and confectioneries. Further studies are planned to identify the determinants of exposure in these workplaces and to assess the concentrations of allergens on the flour dust collected on the filters.

## Figures and Tables

**Table 1:** Characteristics of the bakeries enrolled in the study.

	Craft Bakeries	Industrial Bakeries	All Bakeries
Bakery Type	10	14	24
Employees (*n*)			
<5	8	1	9
5–15	2	9	11
>15	-	4	4
Daily flour consumption (kg)			
<500	10	2	12
500–1000	-	6	6
>1000	-	6	6
Pouring method			
Manual	8	2	10
Pipeline	1	7	8
Pipeline with sleeve	1	5	6

**Table 2:** Levels of flour dust exposure categorized by facility type.

Facility Type	All Samples				Area Samples				Personal Samples			
	N	Mean ± SD (mg/m^3^)	Median (mg/m^3^)	Range (mg/m^3^)	N	Mean ± SD (mg/m^3^)	Median (mg/m^3^)	Range (mg/m^3^)	N	Mean ± SD (mg/m^3^)	Median (mg/m^3^)	Range (mg/m^3^)
Flourmills (*n* = 2)	15	1.525 ± 1.689	0.881 ^a^	0.097–5.642	6	1.744 ± 2.217	0.681	0.156–5.642	9	1.378 ± 1.365	0.881	0.097–4.135
Bakeries (*n* = 3)	26	1.454 ± 1.855	0.603 ^b^	0.043–6.856	13	0.664 ± 0.867	0.323	0.043–3.126	13	2.243 ± 2.252	1.214 ^d^	0.150–6.856
Confectioneries (*n* = 2)	12	1.431 ± 2.038	0.558 ^c^	0.117–7.332	5	1.000 ± 0.989	0.548	0.159–2.656	7	1.739 ± 2.588	0.569	0.117–7.332
Pizzerias (*n* = 2)	5	0.223 ± 0.187	0.127	0.056–0.487	2	0.272 ± 0.305	0.272	0.056–0.487	3	0.190 ± 0.140	0.127	0.093–0.351
All facilities (*n* = 9)	58	1.361 ± 1.772	0.542	0.043–7.332	26	0.948 ± 1.317	0.418	0.043–5.642	32	1.697 ± 2.029	0.856	0.093–7.332

^a^*p* = 0.0160 flour dust levels (all samples) in flourmills vs. levels in pizzerias; ^b^ *p* = 0.0219 flour dust levels (all samples) in bakeries vs. levels in pizzerias; ^c^ *p* = 0.0307 flour dust levels (all samples) in confectioneries vs. levels in pizzerias; ^d^ *p* = 0.0110 personal exposure in bakeries vs. personal exposure in pizzerias (Kruskal–Wallis multiple comparison test and Conover–Iman test).

**Table 3:** Levels of flour dust exposure categorized by bakery type, daily flour consumption and pouring method of mixer tub.

	All Samples				Area Samples				Personal Samples			
	N	Mean ± SD (mg/m^3^)	Median (mg/m^3^)	Range (mg/m^3^)	N	Mean (mg/m^3^)	Median (mg/m^3^)	Range (mg/m^3^)	N	Mean (mg/m^3^)	Median (mg/m^3^)	Range (mg/m^3^)
Bakery Type												
Industrial (*n* = 14)	127	1.944 ± 2.789	0.800	0.043–16.763	70	1.626 ± 2.755	0.462	0.043–16.763	57	2.334 ± 2.805	1.214	0.148–14.055
Craft (*n* = 10)	62	1.540 ± 1.319	1.161	0.062–7.112	35	1.401 ± 1.408	0.741	0.062–6.185	27	1.721 ± 1.195	1.446	0.644–7.112
Daily flour consumption (kg)												
<500	79	1.318 ± 1.261	1.025	0.062–7.112	45	1.181 ± 1.323	0.644	0.062–6.185	34	1.500 ± 1.168	1.240	0.148–7.112
500–1000	48	1.714 ± 2.130	0.789	0.093–7.947	27	1.551 ± 2.272	0.333	0.093–7.667	21	1.923 ± 1.968	0.946	0.298–7.947
>1000	62	2.515 ± 3.400	0.958	0.043–16.763	33	2.056 ± 3.383	0.772	0.043–16.763	29	3.038 ± 3.402	1.948	0.150–14.055
Pouring method												
Manual	55	2.086 ± 2.593	1.214 ^a^	0.062–16.763	32	1.931 ± 3.042	0.879	0.062–16.763	23	2.303 ± 1.836	1.820	0.807–7.122
Pipeline	90	2.206 ± 2.668	1.113 ^b^	0.043–14.055	48	1.836 ± 2.362	0.718	0.043–8.658	42	2.629 ± 2.953	1.866	0.150–14.055
Pipeline with sleeve	44	0.660 ± 0.676	0.408	0.093–3.547	25	0.517 ± 0.569	0.333	0.093–2.640	19	0.848 ± 0.771	0.619	0.148–3.547
All bakeries	189	1.811 ± 2.411	0.946	0.043–16.763	105	1.551 ± 2.386	0.626	0.043–16.763	84	2.137 ± 2.417	1.400 ^c^	0.148–14.055

^a^*p* < 0.0001 pipeline with sleeve vs. manual; ^b^ *p* < 0.0001 pipeline with sleeve vs. pipeline (Kruskal–Wallis multiple comparison test and Conover–Iman test); ^c^ *p* < 0.0001 personal vs. area samples (Mann–Whitney test).

**Table 4:** Inhalable dust exposure categorized by job task in the 24 Italian bakeries.

Job Task	Area Samples				Personal Samples			
	N	Mean ± SD (mg/m^3^)	Median (mg/m^3^)	Range (mg/m^3^)	N	Mean ± SD (mg/m^3^)	Median (mg/m^3^)	Range (mg/m^3^)
Dough making	31	3.002 ± 3.221	2.345	0.176–16.763	29	3.233 ± 2.928	2.345	0.148–14.055
Dough forming	40	1.467 ± 2.044	0.728 ^a^	0.062–8.658	46	1.735 ± 1.999	1.179 ^f^	0.154–12.068
Packaging area	13	0.298 ± 0.242	0.216 ^b,d^	0.043–0.827	9	0.659 ± 0.606	0.349 ^g,h^	0.150–1.851
Baking area	21	0.344 ± 0.195	0.328 ^c,e^	0.093–0.791	-	--	--	--

^a^*p* = 0.0002 dough making vs. dough forming; ^b^ *p* < 0.0001 dough making vs. packaging area; ^c^ *p* < 0.0001 dough making vs. backing area; ^d^ *p* = 0.0002 dough forming vs. packaging area; ^e^ *p* = 0.0004 dough forming vs. backing area; ^f^ *p* = 0.0011 dough making vs. dough forming, personal samples; ^g^ *p* < 0.0001 dough making vs. packaging area, personal samples; ^h^ *p* = 0.0085 dough forming vs. packaging area, personal samples (Kruskal–Wallis multiple comparison test and Conover–Iman test).

**Table 5:** Comparison of changes in flour dust exposure pre-intervention (Phase 2) and post-intervention (Phase 3) in the four Italian bakeries.

	Phase 2				Phase 3			
	N	Mean ± SD (mg/m^3^)	Median (mg/m^3^)	Range (mg/m^3^)	N	Mean ± SD (mg/m^3^)	Median (mg/m^3^)	Range (mg/m^3^)
Total	28	3.352 ± 3.744	1.706	0.093–14.055	28	1.715 ± 1.406	1.360 ^a^	0.156–5.638
Dough making	11	4.864 ± 3.765	4.991	0.254–14.055	11	2.377 ± 1.477	2.002 ^b^	0.741–5.638

^a^*p* = 0.0298 Phase 2 vs. Phase 3; ^b^ *p* = 0.042 Phase 2 vs. Phase 3 (Wilcoxon’s signed ranks test).

## References

[1] Pigatto P.D., Polenghi M.M., Altomare G.F. (1987). Occupational dermatitis in bakers: A clue for atopic contact dermatitis. Contact Dermat..

[2] Brisman J., Torén K., Lillienberg L., Karlsson G., Ahlstedt S. (1998). Nasal symptoms and indices of nasal inflammation in flour-dust-exposed bakers. Int. Arch. Occup. Environ. Health.

[3] Brisman J. (2002). Baker’s Asthma. Occup. Environ. Med..

[4] Karjalainen A., Martikainen R., Klaukka T., Saarinen K., Uitti J. (2003). Risk of asthma among Finnish patients with occupational rhinitis. Chest.

[5] Skjold T., Dahl R., Juhl B., Sigsgaard T. (2008). The incidence of respiratory symptoms and sensitisation in baker apprentices. Eur. Respir. J..

[6] Matsuo H., Uemura M., Yorozuya M., Adachi A., Morita E. (2010). Identification of IgE-reactive proteins in patients with wheat protein contact dermatitis. Contact Dermat..

[7] Page E.H., Dowell C.H., Mueller C.A., Biagini R.E., Heederik D. (2010). Exposure to flour dust and sensitization among bakery employees. Am. J. Ind. Med..

[8] Malo J.-L., Lemiere C., Gautrin D., Labrecque M., Lavoie K. (2009). Asthma and the Workplace.

[9] Ramazzini B. (1713). De Morbis Artificum Diatriba.

[10] Health and Safety Executive (HSE). https://www.hse.gov.uk/statistics/causdis/asthma.pdf.

[11] Ameille J., Pauli G., Calastreng-Crinqu A., Vervloet D., Iwatsubo Y., Popin E., Bayeux-Dunglas M.C., Kopferschmitt-Kub M. (2003). Reported incidence of occupational asthma in France, 1996–99: The ONAP programme. Occup. Environ. Med..

[12] Leira H.L., Bratt U., Slåstad S. (2005). Notified cases of occupational asthma in Norway: Exposure and consequences for health and income. Am. J. Ind. Med..

[13] Wiszniewka M., Walusiak-Skorupa J. (2013). Diagnostic and frequency of work-exacerbated asthma among bakers. Ann. Allergy Asthma Immunol..

[14] Armentia A., Arranz E., Garrote J.A., Santos J. (2015). Wheat as an Allergen: Baker’s Asthma, Food and Wheat Pollen Allergy. Advances in the Understanding of Gluten related Pathology and the Evolution of Gluten-Free Foods.

[15] Money A., Carder M., Agius R. (2017). The Incidence of Work-Related Ill-Health as Reported to the Health and Occupation Research (THOR) Network by Physicians in the Republic of Ireland between 2005 and 2016. https://www.hsa.ie/eng/Workplace_Health/Illness_Reports/ROI_THOR_2017_annual_report.pdf.

[16] Karjalainen A., Leppänen M., Leskinen J., Torvela T., Pasanen P., Tissari J., Miettinen M. (2017). Concentrations and number size distribution of fine and nanoparticles in a traditional Finnish bakery. J. Occup. Environ. Hyg..

[17] Moscato G., Maestrelli P., Bonifazi F., Troise C., Caminati M., Crivellaro M., Olivieri M., Senna G. (2014). OCCUPATION study (OCCUPational Astham: A naTIONal based study): A survey on occupational asthma awareness among italian allergist. Eur. Ann. Allergy Clin. Immunol..

[18] Awad el Karim M.A., Gad el Rag M.O., Omer A.A., El Haimi Y.A. (1986). Respiratory an Allergic Disorders in Workers Exposed to Grain and Flour Dusts. Arch. Environ. Health.

[19] Heederik D., Houba R. (2001). An Exploratory Quantitative Risk Assessment for High Molecular Weight Sensitizer: Wheat Flour. Ann. Occup. Hyg..

[20] Gestis Database. https://limitvalue.ifa.dguv.de/WebForm_ueliste2.aspx.

[21] American Conference of Governmental Industrial Hygienist (ACGIH) (2014). Flour Dust: TLV Chemical Substances.

[22] Scientific Committee on Occupational Exposure Limits (SCOEL) Recommendation from the Scientific Committee on Occupational Exposure Limits for Flour Dust. SCOEL/SUM/123 (2008). http://ec.europa.eu/social/BlobServlet?docId=3869&langId=en.

[23] Houba R., Vanrun P., Heederik D., Doekes G. (1996). Wheat antigen exposure assessment for epidemiological studies in bakeries using personal dust sampling and inhibition ELISA. Clin. Exp. Allergy.

[24] Houba R., Heederik D., Doekes G., E Van Run P. (1996). Exposure-sensitization relationship for alpha-amylase allergens in the baking industry. Am. J. Respir. Crit. Care Med..

[25] Baatjies R., Meijster T., Lopata A., Sander I., Raulf-Heimsoth M., Heederik D., Jeebhay M. (2010). Exposure to Flour Dust in South Africa Supermarket Bakeries: Modelling of Baseline Measurements of an Intervention Study. Ann. Occup. Hyg..

[26] Moghaddasi Y., Mirmohammadi S., Ahmad A., Nejad S.E., Yazdani J. (2014). Health–risk assessment of workers exposed to flour dust: A cross–sectional study of random samples of bakeries workers. Atmos. Pollut. Res..

[27] Kirkeleit J., Hollund B.E., Riise T., Eduard W., Bråtveit M., Storaas T. (2016). Bakers’ Exposure to Flour Dust. J. Occup. Environ. Hyg..

[28] Viegas C., Fleming G.T.A., Kadir A., Almeida B., Caetano L.A., Gomes A., Twarużek M., Kosicki R., Viegas S., Coggins M.A. (2020). Occupational Exposures to Organic Dust in Irish Bakeries and a Pizzeria Restaurant. Microorganisms.

[29] Brisman J., Järvholm B., Lillienberg L. (2000). Exposure-response relations for self reported asthma and rhinitis in bakers. Occup. Environ. Med..

[30] Houba R., Heederik D., Doekes G. (1998). Wheat Sensitization and Work-Related Symtons in the Baking Industry are Preventable. An Epidemiologic Study. Am. J. Respir. Crit. Care Med..

[31] Meijster T., Tielemans E., Schinkel J., Heederik D. (2008). Evaluation of Peak Exposures in the Dutch Flour Processing Industry: Implications for Intervention Strategies. Ann. Occup. Hyg..

[32] Meijster T., Tielemans E., De Pater N., Heederik D. (2007). Modelling Exposure in Flour Processing Sectors in The Netherlands: A Baseline Measurement in the Context of an Intervention Program. Ann. Occup. Hyg..

[33] Nij E.T., Hilhorst S., Spee T., Spierings J., Steffens F., Lumens M., Heederik D. (2003). Dust control measures in the construction industry. Ann. Occup. Hyg..

[34] Baatjies R., Meijster T., Heederik D., Sander I., Jeebhay M.F. (2014). Effectiveness of interventions to reduce flour dust exposures in supermarket bakeries in South Africa. Occup. Environ. Med..

[35] Health and Safety Executive (HSE). https://www.fob.uk.com/wp-content/uploads/2018/07/BAKERS-DOZEN-MASTER-DEC-2014-July-2018-amendments.pdf.

[36] Burstyn I., Teschke K., Kennedy S.M. (1997). Exposure levels and determinants of inhalable dust exposure in bakeries. Ann. Occup. Hyg..

[37] Stobnicka A., Górny R.L. (2015). Exposure to flour dust in the occupational environment. Int. J. Occup. Saf. Ergon..

[38] Fishwick D., Harris-Roberts J., Robinson E., Evans G., Barraclough R., Sen D., Curran A.D. (2011). Impact of worker education on respiratory symptoms and sensitization in bakeries. Occup. Med..

[39] Health and Safety Executive (HSE). https://www.hse.gov.uk/asthma/bakers.htm.

[40] Heederik D., Henneberger P.K., Redlich C.A. (2012). Primary prevention: Exposure reduction, skin exposure and respiratory protection. Eur. Respir. Rev..

